# Impact of School Nurses on Children with Food Allergies: A Comprehensive Review

**DOI:** 10.3390/children12020201

**Published:** 2025-02-08

**Authors:** Silvio Simeone, Greta Aquilone, Caterina Mercuri, Flavia Lotito, Vincenzo Bosco, Teresa Rea, Roberto Berni Canani, Rita Nocerino

**Affiliations:** 1 Department of Clinical and Experimental Medicine, University of Catanzaro MagnaGraecia, 88100 Catanzaro, Italy; c.mercuri@unicz.it; 2 Department of Translational Medical Science, University of Naples Federico II, 80138 Naples, Italy; gr.aquilone@studenti.unina.it (G.A.); berni@unina.it (R.B.C.); rita.nocerino@unina.it (R.N.); 3 Faculty of Medicine, Master’s Degree Course in Nursing and Midwifery Sciences, University Nostra Signora del Buon Consiglio, 1000 Tirana, Albania; f.lotito10590@stud.unizkm.al; 4 Department of Medical and Surgical Sciences, University Hospital Mater Domini, Magna Graecia University, 88100 Catanzaro, Italy; vincenzo.bosco@unicz.it; 5 Department of Public Health, University of Naples Federico II, 80138 Naples, Italy; teresa.rea@unina.it; 6 Immunonutrition Lab at CEINGE Advanced Biotechnologies, University of Naples Federico II, 80138 Naples, Italy; 7 Department of Biomedicine and Prevention, University of Rome “Tor Vergata”, 00133 Rome, Italy

**Keywords:** food allergy management, school nurses, anaphylaxis prevention, pediatric health, emergency preparedness, psychosocial support, individualized health plans (IHPs), epinephrine auto-injectors, inclusive education, allergy safety policies

## Abstract

Background. Food allergies (FAs) are a significant public health concern, affecting 6–8% of children worldwide, with a growing prevalence. Schools are high-risk environments for allergic reactions, including anaphylaxis, which can be life-threatening. Alarmingly, up to 16–18% of children with FAs experience allergic reactions at school, often due to accidental exposure. Additionally, up to 25% of anaphylactic reactions in schools occur in children with no prior diagnosis of FA, emphasizing the critical need for school-wide preparedness and robust emergency action plans. School nurses play a pivotal role in managing FAs through individualized health plans, emergency preparedness, staff training, and psychosocial support. This review aims to evaluate the multifaceted role of school nurses in ensuring the safety, health, and psychosocial well-being of children with FAs. It also seeks to identify systemic challenges and gaps in allergy management to inform targeted interventions and future research. Methods. This comprehensive review synthesizes evidence on the role of school nurses in FA management. A systematic literature search was conducted across PubMed, CINAHL, Scopus, and Cochrane, targeting studies published between 2014 and 2024. The search identified 6313 articles, of which 5490 remained after duplicate removal. After title and abstract screening, 60 articles were selected for full-text evaluation, with 59 included in the final review. Thematic analysis identified six domains: preventive measures, emergency preparedness, communication, health outcomes, psychosocial support, and systemic challenges. Results. The review highlights the critical contributions of school nurses to FA management. They improve safety by implementing Individualized Health Plans (IHPs) and Emergency Action Plans (EAPs), ensuring timely administration of epinephrine and reducing delays during emergencies. Preventive strategies, such as allergen-free zones and comprehensive training for staff, minimize exposure risks. Psychosocial interventions led by nurses alleviate stigma, bullying, and anxiety, enhancing the quality of life for children with FAs. Despite these benefits, barriers persist, including insufficient nurse-to-student ratios, limited access to emergency resources like stock epinephrine, and disparities in allergy management across socioeconomic and geographic contexts. Conclusions. School nurses are integral to managing FAs, ensuring safety, fostering inclusion, and addressing psychosocial needs. Addressing systemic barriers and ensuring equitable resource distribution are essential to optimize their impact. Future research should focus on the long-term outcomes of nurse-led interventions, strategies to reduce disparities, and the potential role of digital tools in improving allergy management.

## 1. Introduction

Food allergies (FAs) are a significant and growing public health concern, particularly among pediatric populations worldwide. It is estimated that 6–8% of children globally suffer from FAs, and this prevalence has increased significantly over the past two decades [1,2,3]. The most common allergens include peanuts, tree nuts, milk, eggs, soy, wheat, fish, and shellfish, which account for over 90% of food-induced allergic reactions [4,5,6]. Severe allergic reactions, including anaphylaxis, can occur unpredictably and require immediate intervention [7,8]. Alarmingly, up to 16–18% of children with FAs experience allergic reactions while at school, underscoring the need for preventive measures and preparedness in educational settings [9,10,11].

Fatal food-induced anaphylaxis often results from delays in administering epinephrine, the primary treatment for severe allergic reactions [12]. In fact, up to 25% of anaphylactic reactions in schools occur in children with no prior diagnosis of FA, highlighting the importance of school-wide readiness and emergency action plans [13,14]. Additionally, the psychosocial burden of FAs cannot be overlooked. Children often face anxiety, stigma, bullying, and exclusion, which may negatively impact their emotional well-being and academic experience [15]. Parents too experience significant stress due to communication gaps and inconsistent allergy management policies in schools [16,17].

School nurses are pivotal in managing the safety and health of children with FAs in school settings [4,18,19]. As primary healthcare providers within the educational environment, they develop and implement Individualized Health Plans (IHPs), train school personnel to recognize and respond to allergic reactions, and ensure timely administration of life-saving medications, such as epinephrine auto-injectors [4,18,19]. The presence of well-trained school nurses significantly improves preparedness, reduces delays in treatment, and minimizes adverse outcomes during allergic emergencies [20,21,22].

School nurses also play an essential role in the education and advocacy of students with FAs, their peers, parents, and school staff [23,24]. In fact, they educate staff, students, and parents about allergen avoidance, symptom recognition, and emergency protocols, fostering collective adherence to allergy management plans [23,24,25]. Schools with dedicated nurses report improved health outcomes, reduced allergic incidents, and enhanced psychosocial well-being for children with FAs as compared to schools without dedicated nurses or those with limited access to trained medical professionals [26,27]. Despite their critical role, barriers persist, including insufficient nurse-to-student ratios, limited resources for allergy training, and inconsistent implementation of FA guidelines [28,29].

In rural and socioeconomically disadvantaged schools, disparities in access to epinephrine auto-injectors and emergency preparedness further exacerbate the risks for children with FAs [30,31]. Addressing these challenges requires systemic changes, including targeted educational interventions, stock epinephrine policies, and improved collaboration among healthcare providers, schools, and families [32,33].

## 2. Materials and Methods

### 2.1. Objectives

The objective of this comprehensive review is to evaluate the role and the impact of school nurses on the safety, health, and overall well-being of children with FAs. Specifically, the review will explore the role of school nurses in preventing allergen exposure, managing allergic emergencies, and addressing the psychosocial challenges faced by affected children and their families. By synthesizing current evidence, this review aims to highlight the critical importance of school nurses in creating safe, supportive, and inclusive school environments for children with FAs and to identify gaps for future research.

### 2.2. Search Strategy

The literature search was conducted using four electronic databases: PubMed, CINAHL, Scopus, and Cochrane. The following search string was applied: “school nurse OR school health nurse OR school nursing OR school health nursing AND food allergy OR food allergies AND children OR adolescents OR youth OR child OR teenager”.

The search targeted articles published in English between 2014 and 2024. Only studies focusing on school nurses’ roles in managing FAs in school settings were included. The search process was conducted from August 2024 to November 2024, and all identified records were imported into Rayyan (Rayyan Systems Inc., Cambridge, MA, USA), a web-based tool for systematic review management.

### 2.3. Inclusion and Exclusion Criteria

Inclusion criteria:

- Articles published in English.
- Studies conducted between 2014 and 2024.
- Research focusing on school nurses or health nurses in school contexts and their involvement with children or adolescents with FAs.
- Peer-reviewed articles of any study design, including systematic reviews, observational studies, and intervention studies.

Exclusion criteria:

- Studies unrelated to school-based interventions or not involving school nurses.
- Non-peer-reviewed materials, conference abstracts, and opinion pieces.

### 2.4. Selection Process

**Figure 1:**
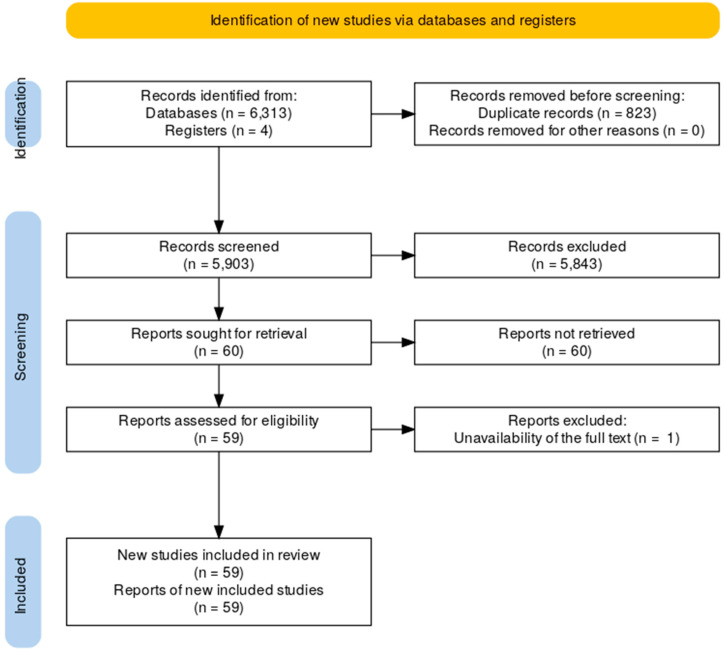
Flow diagram illustrating the selection process.

The initial search identified 6313 articles. After removing 823 duplicates, 5490 articles remained for title and abstract screening. Two authors (R.N. and F.L.) independently screened these records to ensure consistency in the inclusion process. Disagreements were resolved through consensus discussions. A total of 60 articles were selected for full-text evaluation, of which 1 was excluded due to unavailability of the full text. The final review included 59 studies. A flow diagram illustrating the selection process was constructed to provide a transparent overview (Figure 1).

### 2.5. Data Analysis

The methodology used for the analysis in this review involved several steps to ensure the reliability and consistency of the findings [34,35]. First, data were coded and categorized independently by two reviewers (R.N. and F.L.) to identify recurring themes and patterns in the literature. This dual-review process minimized bias and ensured a comprehensive evaluation of the included studies. Discrepancies between reviewers were resolved through discussion and consensus.

The analysis identified six primary domains that define the multifaceted role of school nurses in managing FAs, emphasizing their critical contributions to ensuring safety, health, and psychosocial well-being. These domains were derived from a thematic synthesis of the included studies, providing a comprehensive framework for understanding the responsibilities and challenges faced by school nurses. The domains are as follows:

1. Preventive measures
2. Emergency preparedness
3. Communication and coordination
4. Impact on health outcomes for children with FAs
5. Psychosocial support and education
6. Challenges in allergy management

By organizing findings into these domains, the thematic analysis provided a structured framework to explore both the contributions of school nurses and the systemic challenges affecting their capacity to manage FAs effectively. The findings were organized to highlight the comprehensive scope of school nurses’ responsibilities, as well as the systemic and logistical barriers that impact their ability to provide optimal care.

## 3. Results

### 3.1. Preventive Measures

School nurses play a pivotal role in implementing preventive strategies to manage FAs and ensure the safety of children in educational settings. One of their primary responsibilities is the development of Individualized Health Plans (IHPs) and Emergency Action Plans (EAPs), tailored to the specific needs of each allergic child. These plans outline strategies for allergen avoidance, symptoms to monitor, and emergency protocols to ensure effective responses [5,19].

A crucial element of these measures is training school personnel, students, and parents to recognize common allergens, avoid cross-contamination, and promptly identify symptoms of allergic reactions, enabling timely intervention [11,23].

Additionally, nurses promote safe practices such as creating “allergy-safe zones” in cafeterias, classrooms, and shared spaces, minimizing accidental exposures. These safe zones, combined with proactive planning and risk assessments, significantly reduce the likelihood of allergic incidents in schools [2,3].

Educational programs led by nurses increase staff preparedness and confidence in managing FAs, ensuring safety in resource-limited or rural schools where medical support may be scarce [13,36,37]. Furthermore, nurses advocate for policies such as “no sharing” rules and safe food preparation protocols to mitigate exposure risks [38]. Studies highlight the importance of consistent allergy education and digital tools, which enhance awareness and preparedness across the entire school community [33].

Regular training sessions for school staff on allergy management and emergency response significantly improve preparedness and confidence in handling allergic reactions [39]. Mandatory training programs serve as a model, equipping educators and non-medical staff with the skills to administer epinephrine and recognize symptoms of anaphylaxis. These initiatives not only enhance the safety of allergic students but also reduce delays in emergency interventions, particularly in schools with limited nurse availability [39].

### 3.2. Emergency Preparedness

Emergency preparedness is a cornerstone of FA management, and school nurses play an indispensable role in ensuring schools are equipped to respond effectively to allergic emergencies [8,31,40]. Immediate administration of epinephrine auto-injectors (EAIs) during anaphylaxis is critical for preventing severe outcomes and fatalities, as delays in treatment can significantly increase risks [4,41]. Nurses ensure that epinephrine auto-injectors are readily available in accessible locations throughout the school and that staff are properly trained to use them effectively [23,42].

The implementation of stock epinephrine policies, which allow schools to maintain unassigned epinephrine auto-injectors for use in first-time or undiagnosed allergic reactions, is a critical safety measure [10,43]. Simulation drills, regular anaphylaxis response training, and established emergency protocols are integral components of preparedness, enabling school personnel to recognize symptoms promptly, administer epinephrine, and activate emergency medical services [25,31,44].

Despite these advancements, barriers such as inconsistent staff training and lack of funding for emergency resources persist, particularly in schools with limited nurse availability. Nurses advocate for legislative support and increased funding to address these gaps and ensure comprehensive readiness across all schools [11,45].

### 3.3. Communication and Coordination

Effective communication and coordination between school nurses, teachers, and healthcare providers are essential to ensure continuity of care and safety for children with FAs. Nurses act as liaisons to facilitate clear communication regarding a child’s allergy status, emergency action plans, and any updates from healthcare providers [3,19]. For instance, Järvenpää et al. (2014) [46] demonstrated that school nurses play a pivotal role in interviewing parents to collect precise data on physician-diagnosed FAs. This ensures that management plans are based on reliable information and tailored to the specific needs of each child. By working closely with parents, nurses ensure that allergy management plans are up to date and that all necessary medications and resources are available at school [18,45].

Coordination extends to training cafeteria staff, teachers, and administrators to implement proper allergen labeling, minimize cross-contact during food preparation, and create inclusive environments for students with FAs [25,29,33,47]. Nurses also educate peers and teachers on fostering empathy and inclusion, addressing social stigma, and supporting the emotional well-being of children with allergies [48,49].

Through collaboration with pediatricians and allergists, nurses ensure that schools have the latest guidelines on FA management. Open communication among stakeholders enhances preparedness and alleviates anxiety for both children and their families [7,50,51].

### 3.4. Impact on Health Outcomes for Children with FAs

School nurses play a fundamental role in improving safety and reducing the frequency and severity of allergic reactions in children with FAs. By developing IHPs and EAPs, they ensure that protocols for allergen avoidance and emergency responses are clear and actionable. Schools with such plans have reported significantly fewer allergy-related incidents, owing to enhanced preparedness and better training of staff and students [36].

Stock epinephrine availability has been identified as a critical safety measure, particularly for first-time anaphylactic events in children without prior diagnoses of FA [52]. Regular staff training sessions conducted by school nurses improve preparedness and response times during emergencies, which are essential to preventing severe outcomes [53].

In addition to emergency preparedness, nurses implement preventive strategies such as allergen-free zones, cross-contact prevention protocols, and food preparation monitoring. These interventions significantly reduce accidental exposures in shared spaces like cafeterias and classrooms [9]. Moreover, nurse-led programs emphasizing proper handwashing and food labeling practices create a safer environment for children with FAs [11].

Recent technological advancements, including eHealth tools and digital training modules, have further strengthened staff and student awareness, fostering environments that are both safe and inclusive [33].

### 3.5. Psychosocial Support and Education

#### 3.5.1. Psychosocial Benefits

FA can profoundly affect the emotional and social well-being of children, often leading to anxiety, stigma, and isolation. School nurses mitigate these effects by fostering supportive and inclusive environments. Their advocacy for empathy and understanding among peers and staff helps reduce bullying and promote social inclusion. For instance, peer education programs led by nurses have been shown to decrease stigma and create a more inclusive school culture [54]. The emotional impact of FAs on children and their families is profound, often leading to heightened anxiety and social exclusion. School nurses play a pivotal role in addressing these challenges by fostering supportive environments and advocating for inclusive policies [55].

The constant risk of allergic reactions often results in heightened anxiety for children and their families. Nurses alleviate these concerns by ensuring robust safety measures are in place, which reassures parents and instills confidence in the child’s ability to participate in school activities [22]. Initiatives such as allergy-safe zones and anti-bullying policies, supported by nurse-led interventions, significantly improve children’s emotional resilience and sense of belonging [56]. For example, peer education programs led by nurses can reduce stigma and promote empathy, creating a more welcoming atmosphere for allergic students [55]. In addition, the training program described by White et al. (2016) [57] significantly increased awareness among school staff regarding the bullying risks faced by students with FAs. This enhanced understanding encourages early intervention and promotes a more inclusive school environment.

Additionally, nurses provide emotional support by maintaining open communication with families and staff, fostering trust and collaboration. By maintaining open communication with parents and staff, nurses also alleviate parental concerns, building trust and confidence in the school’s ability to manage allergies effectively [55]. This comprehensive approach improves the overall quality of life for children with FAs and their caregivers [58].

#### 3.5.2. Role in Education and Self-Management

School nurses are instrumental in empowering children to manage their allergies independently. Through age-appropriate education, they teach children to identify allergens, recognize symptoms of allergic reactions, and use epinephrine auto-injectors correctly. White et al. (2016) [57] demonstrated that school nurses are key facilitators of educational initiatives in allergy management. Their study highlighted the implementation of a computer-based training module that significantly improved school staff’s knowledge, confidence, and preparedness in handling food allergy emergencies. This initiative not only addressed clinical aspects, such as recognizing symptoms and administering epinephrine, but also tackled psychosocial elements, including reducing stigma and fostering empathy among students and staff. This education fosters self-advocacy and promotes independence, equipping children with the skills needed for lifelong allergy management [53].

Educational initiatives extend beyond the students to include parents, teachers, and staff. Nurses conduct workshops and training sessions focused on self-management strategies, such as reading food labels, avoiding cross-contact, and communicating effectively about allergy needs. These programs enhance confidence and knowledge among children, enabling them to participate safely in various school and extracurricular activities [20].

Collaboration between school nurses, healthcare providers, families, and educators ensures consistent and effective allergy management. This interprofessional approach not only supports the child’s self-management journey but also reinforces the importance of adhering to allergy protocols at school and home, ensuring long-term health outcomes [3].

By integrating safety, psychosocial support, and education, school nurses play an indispensable role in improving health outcomes, reducing risks, and fostering resilience in children with FAs. Their contributions extend beyond the physical aspects of allergy management, addressing the emotional and educational needs of students in a comprehensive and impactful manner.

### 3.6. Challenges Faced by School Nurses in Allergy Management

#### 3.6.1. Resource Limitations

School nurses frequently encounter significant resource challenges, which hinder their ability to manage FAs effectively. High nurse-to-student ratios remain a critical issue, with some nurses responsible for multiple schools, particularly in rural or socioeconomically disadvantaged areas. This limits their ability to provide individualized care and respond promptly to emergencies [3,54]. In some countries, the role of a school nurse is not a standardized provision, leaving schools without a dedicated healthcare professional to manage allergy-related emergencies and preventive measures. Funding limitations exacerbate the problem, making it difficult for schools to maintain a supply of stock epinephrine auto-injectors and implement comprehensive training programs for staff [22,36].

Programs such as EPIPEN4SCHOOLS have provided essential resources, but their reach is limited, particularly in underfunded districts. Research highlights that schools with insufficient funding struggle to create allergen-safe zones or conduct regular anaphylaxis drills, leaving students at greater risk of adverse outcomes [33,52]. The lack of accessible training materials and resources also impacts the ability of school staff to recognize and respond to allergic reactions effectively [9]. Additionally, disparities in access to allergy management resources, such as stock epinephrine and specialized training, are particularly pronounced in rural and socioeconomically disadvantaged schools. Uhm et al. (2020) [55] emphasized the need for targeted interventions to address these inequities, including the implementation of digital tools and telehealth consultations. Ensuring equitable resource distribution is essential for safeguarding the health and well-being of all students with FAs, regardless of their geographic or economic background.

#### 3.6.2. Legal and Policy Constraints

Legal and policy barriers add complexity to allergy management in schools. State and local policies regarding the availability and administration of epinephrine vary widely, resulting in inconsistent practices across districts. Some states mandate stock epinephrine, while others leave the decision to individual schools, creating gaps in emergency preparedness [13,23,59]. Furthermore, liability concerns discourage school staff from administering epinephrine or participating in allergy management protocols, even when trained [60].

The absence of uniform guidelines for FA management contributes to disparities in care. For example, policies governing allergen-free zones or food labeling protocols are often inconsistently applied, leaving children at risk of accidental exposures [16,32]. Systemic barriers, such as the absence of uniform guidelines and inadequate training programs, further exacerbate these challenges. A mixed-methods review by Uhm et al. (2020) [55] identified gaps in staff knowledge and the lack of interdisciplinary communication as key obstacles. For instance, school nurses frequently rely on parents for daily management decisions due to limited coordination with primary care providers. Addressing these barriers requires a systemic approach, including standardized training modules and stronger partnerships between schools and healthcare providers. Additionally, the administrative burden of maintaining IHPs and EAPs consumes valuable time that could otherwise be spent on direct care [3].

#### 3.6.3. Knowledge Gaps and Training Needs

Despite their critical role, many school nurses and staff lack comprehensive training in FA management. Studies show that gaps in knowledge about anaphylaxis symptoms and emergency response protocols hinder effective care. For example, non-nursing staff often lack the confidence to administer epinephrine, delaying life-saving interventions during emergencies [61,62].

School nurses also report a need for specialized training to address complex cases, such as managing multiple allergens or providing psychosocial support to children with FAs [41]. Digital platforms and simulation-based training programs have proven effective in improving preparedness and confidence among school personnel [22,33]. However, these programs are not universally available, particularly in resource-constrained districts. Efforts to address these gaps include interdisciplinary training sessions and collaborative initiatives between healthcare providers and educational institutions. Regular updates to evidence-based guidelines and the development of standardized resources are critical to equipping school nurses with the tools they need to support children with FAs comprehensively [49,59].

## 4. Discussion

The pivotal role of school nurses in managing FAs is well-documented, highlighting their contributions to improving safety, reducing the frequency and severity of allergic reactions, and fostering inclusive environments. Nurse-led interventions, such as the implementation of IHPs and EAPs, significantly enhance preparedness and ensure timely administration of life-saving medications like epinephrine auto-injectors [36,52]. These measures reduce delays in emergency responses and improve safety outcomes, particularly in schools with established protocols and stock epinephrine availability [22,63].

Beyond physical safety, school nurses address psychosocial challenges by reducing anxiety, stigma, and bullying [61,64,65]. Their advocacy fosters empathy among peers and creates supportive environments for children with FAs [54]. Educational initiatives led by nurses enhance awareness and preparedness among school staff, ensuring better management of allergy-related emergencies [61]. However, persistent challenges—such as high nurse-to-student ratios, resource limitations, and inconsistent training—undermine the full potential of these interventions, particularly in underfunded and rural schools [3,62].

Compared to teacher training and parent education, school nurses provide a uniquely specialized and systematic approach to managing FAs. Teacher training programs focus on allergen awareness and emergency responses but often lack the clinical expertise necessary for medical interventions during anaphylactic emergencies [3]. Parent education ensures continuity of care at home but may not address the complex challenges children face in school settings, where professional medical oversight is critical [18,59].

The inclusion of school nurses as part of a multidisciplinary approach bridges these gaps, providing a coordinated effort between families, educators, and healthcare systems. Programs like EPIPEN4SCHOOLS illustrate the effectiveness of nurse-led advocacy in implementing stock epinephrine policies and comprehensive allergy management protocols, which significantly improve emergency response outcomes compared to schools relying solely on teacher or parent interventions [22,33].

To maximize the impact of school nurses in FA management, several policy and practice recommendations have been proposed by the Centers for Disease Control and Prevention (CDC) and the National Association of School Nurses (NASN) [65,66]. First, increasing funding to improve nurse-to-student ratios and ensure the availability of critical resources like epinephrine auto-injectors is essential. Policymakers should mandate the presence of full-time nurses in schools, standardize training protocols, and require all educational institutions to stock epinephrine to ensure uniform preparedness [32,54].

The implementation of comprehensive policies, such as the Chicago Public Schools (CPS) Emergency EpiPen Policy, highlights the critical role of legislative frameworks in supporting allergy management. This policy not only ensures the availability of stock epinephrine auto-injectors but also includes liability protections for school nurses administering treatment during emergencies [39]. However, funding limitations and inconsistent nurse coverage underscore the need for enhanced resource allocation and policy enforcement to ensure equity in allergy care across all schools [39].

Addressing knowledge gaps through digital learning tools and simulation-based training can enhance preparedness and confidence among school staff, fostering a cohesive and well-trained workforce capable of managing allergy-related emergencies [22,33]. Legal protections for school personnel administering epinephrine in emergencies are also necessary to mitigate liability concerns and encourage timely interventions [10].

Collaboration between schools, healthcare providers, and community organizations is critical to developing standardized guidelines and promoting interprofessional coordination. Such partnerships can ensure consistent application of best practices in allergy management, creating safer and more inclusive environments for students with FAs [59].

By addressing systemic barriers and enhancing support structures, school systems can empower nurses to optimize FA management, safeguarding the health and well-being of affected children while promoting an inclusive educational experience.

## 5. Conclusions

School nurses play an essential role in managing FAs, offering both immediate clinical interventions and long-term preventive strategies that significantly enhance the safety and quality of life for children. By implementing IHPs and EAPs, they reduce the incidence and severity of allergic reactions in schools, ensuring preparedness for emergencies [36,52]. Their advocacy extends to educating school staff, students, and families about allergen avoidance, symptom recognition, and the correct administration of life-saving medications, such as epinephrine auto-injectors, fostering a culture of inclusion and safety [19,20].

Moreover, the psychosocial benefits of having trained nurses include reduced stigma, bullying, and anxiety among food-allergic children, allowing for better social and academic integration [41,54]. Despite these successes, challenges such as resource limitations, variability in policy implementation, and gaps in staff training remain substantial barriers, especially in underfunded and rural schools [3,33].

## 6. Future Research Directions

Further research is needed to explore the long-term impacts of nurse-led interventions on the health outcomes and psychosocial well-being of children with FAs. Longitudinal studies could examine the effectiveness of preventive measures, such as allergen-free zones and stock epinephrine policies, on reducing allergic incidents and improving emergency responses [22,23]. Additionally, investigating disparities in allergy management across socioeconomic and geographic contexts could identify targeted strategies to bridge these gaps [29,30].

The integration of technology, such as digital training platforms and eHealth tools, warrants further exploration for its potential to enhance education, preparedness, and collaboration among school staff and healthcare providers [33]. Finally, studying the cost-effectiveness of various school-based interventions could provide valuable insights for policymakers, helping to prioritize resources for maximizing safety and inclusivity in educational settings [32,54].
